# SEM Evaluation of Surface Wear on Drills from Selected Implant Systems—In Vitro Study

**DOI:** 10.3390/ma19040669

**Published:** 2026-02-10

**Authors:** Piotr Kosior, Kamila Wiśniewska, Natalia Struzik, Michał Kulus, Edward Chlebus, Agata Małyszek, Klaudia Sztyler, Jacek Matys, Maciej Dobrzyński

**Affiliations:** 1 Department of Conservative Dentistry with Endodontics, Wroclaw Medical University, Krakowska 26, 50-425 Wroclaw, Poland; 2 Dental Surgery Department, Wroclaw Medical University, Krakowska 26, 50-425 Wroclaw, Poland; kamila.wisniewska@umw.edu.pl; 3 Department of Nutrition and Drug Research, Faculty of Health Sciences, Institute of Public Health, Jagiellonian University Medical College, Skawińska 8, 31-066 Krakow, Poland; natalia.struzik98@gmail.com; 4 Division of Ultrastructural Research, Wroclaw Medical University, Chałubińskiego 6a, 50-368 Wrocław, Poland; michal.kulus@umw.edu.pl; 5 Faculty of Mechanical Engineering, Wroclaw University of Science and Technology, Lukasiewicza 5, 50-371 Wroclaw, Poland; edward.chlebus@pwr.edu.pl; 6 Department of Biostructure and Animal Physiology, Wrocław University of Environmental and Life Sciences, Kozuchowska 1, 51-631 Wroclaw, Poland; agata.malyszek@upwr.edu.pl; 7 Department of Pediatric Dentistry and Preclinical Dentistry, Wroclaw Medical University, Krakowska 26, 50-425 Wroclaw, Poland; klaudia.sztyler@umw.edu.pl (K.S.); maciej.dobrzynski@umw.edu.pl (M.D.)

**Keywords:** cutting surface deformation, dental implant drills, drill surface wear

## Abstract

Purpose: The aim of this in vitro study was to evaluate the degree of surface wear in implant drills from four commercial systems subjected to standardized osteotomy cycles. Materials: Four implant systems (Osstem, Megagen, Straumann, and Bego) were evaluated using sets of three drills of increasing diameters. A total of 120 osteotomies were performed in standardized porcine rib specimens under controlled drilling conditions (1200 rpm, continuous 4 °C saline irrigation, 32:1 reduction handpiece). After each drilling series, drills were cleaned, sterilized, and analyzed using SEM in three orientations. Wear was assessed using a seven-parameter scoring system. Multifactorial ANOVA, Pearson correlation, and hierarchical clustering were employed to evaluate the effects of drill brand, diameter, and wear patterns. Results: Both drill brand and diameter significantly influenced total wear scores (*p* < 0.001). Small-diameter pilot drills exhibited the highest wear, while large-diameter drills showed minimal degradation. Among the systems tested, Bego drills demonstrated the greatest overall wear, whereas Osstem drills—particularly the 2.0 mm drill—displayed unusually low wear for their size. A strong negative correlation between drill diameter and wear score was observed. Cluster analysis identified distinct wear patterns associated with specific drill sizes, with small drills showing prominent guide-face nicks and accumulation formation, medium drills exhibiting chipping and rake angle cleavage, and large drills presenting minimal wear. SEM imaging confirmed progressive surface deterioration, including edge rounding, microchipping, and irregular surface defects. Conclusions: Implant drill wear is strongly dependent on drill diameter, and cutting geometry. Small-diameter drills are most susceptible to surface degradation, which may increase friction and thermal load during osteotomy. Systems with enhanced material properties or optimized geometries demonstrated superior wear resistance. These findings highlight the importance of monitoring drill condition, adhering to recommended reuse limits, and considering advanced drill coatings or materials to ensure safe and predictable implant site preparation. Further research incorporating real-time thermal measurements and extended drilling cycles is needed to establish evidence-based guidelines for drill longevity and clinical performance.

## 1. Introduction

The effectiveness of implantology procedures depends on the careful preparation of the bone bed, which is critical for achieving primary stability and long-term osseointegration [1,2]. Various osteotomy techniques exist, including rotary systems, ultrasound devices, laser methods, and manual approaches [3,4,5]; however, rotary systems remain the most frequently used due to their efficiency and predictable outcomes. These systems employ progressively larger drills to create osteotomies while preserving bone structure [6,7]. Implantological drills are therefore essential instruments in forming precise cylindrical preparations that support successful implant placement [8,9]. During repeated clinical use, however, drill materials inevitably undergo wear, resulting from mechanical loading and friction during osteotomy [8,9,10,11]. Common drill materials include stainless steel, cobalt–chromium alloys, and more recently titanium-based alloys, each offering different balances of strength, durability, and biocompatibility [12,13,14,15,16,17]. Stainless steel is cost-effective but more prone to wear than cobalt–chromium or titanium alloys. Mechanical wear manifests as microabrasions, edge rounding, and loss of sharpness, potentially reducing osteotomy precision and impairing clinical outcomes [9,18,19].

Bone quality can be quantitatively evaluated using computed tomography through analysis of Hounsfield units (HU), a method first proposed by Misch in 1999 [20]. This classification system describes a continuum of bone density ranging from highly mineralized cortical bone to low-density trabecular bone, categorized as D1 (>1250 HU), D2 (850–1250 HU), D3 (350–850 HU), D4 (150–350 HU), and D5 (<150 HU) [20]. Bone density is a critical determinant of implant site preparation, as it directly influences cutting resistance, heat generation, and drilling efficiency [21]. Moreover, adequate bone density is essential for achieving high primary implant stability, which is a prerequisite for successful immediate or early loading protocols and for reducing the risk of micromotion during the initial healing phase [21]. Material degradation is influenced by drilling speed, torque, axial load, irrigation, and bone density [9]. Among these parameters, drilling speed plays a particularly important role, as it directly affects cutting efficiency, frictional heat generation, and mechanical interaction at the drill–bone interface [8,9]. Although manufacturers provide diameter-specific rotational speed recommendations (most often in the range of 800 to 1500 rpm, depending on drill diameter) to optimize performance, the relationship between drilling speed, heat generation, and drill wear remains complex and is strongly influenced by drilling force, irrigation, and drilling time. Previous experimental studies have demonstrated that neither very low nor excessively high rotational speeds are optimal, as both conditions may increase frictional effects and thermal load during osteotomy [10,22]. Also, dense cortical bone generates higher friction forces and accelerates wear, whereas trabecular bone causes comparatively less surface damage [22,23,24]. Adequate irrigation reduces heat at the drill–bone interface, mitigating both thermal injury and drill degradation [25,26,27]. Insufficient cooling can increase frictional heat and compromise biological responses. Drill wear affects performance by increasing preparation time, requiring higher penetration forces, and elevating temperature—potentially exceeding thresholds for bone necrosis, impairing cellular viability, and jeopardizing osseointegration [28,29,30,31,32,33,34,35]. Regular inspection, controlled reuse, and timely replacement of drills are therefore essential to minimize thermomechanical complications. Improvements in surface engineering, such as diamond-like carbon or titanium nitride coatings, have shown promise in reducing wear and extending drill longevity [11,17,36]. Nonetheless, wear remains unavoidable due to clinical loading conditions and bone characteristics [8,12,22,23].

Despite the routine reuse of implant drills, no universal guideline defines their safe operational lifespan. Wear progression varies with drill system, material, geometry, and the number of osteotomies and sterilization cycles [8,23,37,38]. Existing studies mostly examine temperature generation or drilling efficiency, while fewer assess microstructural surface degradation under standardized conditions [9]. This lack of comparative data limits clinicians’ ability to determine when a drill should be replaced to avoid excessive heat, reduced cutting precision, and compromised bone viability. Understanding how drill design, material properties, and mechanical loading influence wear progression is therefore essential, especially in modern implantology, which emphasizes minimally invasive techniques, thermal control, and bone preservation to enhance osseointegration and long-term outcomes [8].

This comparative analysis was not intended to guide implant system selection, but to provide standardized information on drill wear behavior that may support clinicians in monitoring drill condition, optimizing reuse protocols, and minimizing thermal and mechanical risks during osteotomy. The aim of the present study was to evaluate the degree and pattern of wear on the working surfaces of implant drills used for bone bed preparation. To achieve this, drills from four implant systems were subjected to identical drilling cycles under laboratory conditions simulating clinical parameters, enabling a comparative assessment of surface degradation and its potential implications for drilling efficiency and thermal safety.

## 2. Materials and Methods

### 2.1. Study Design

This in vitro experimental study was designed to assess the degree and nature of surface wear on implant drills originating from different commercial systems and subjected to standardized drilling protocols. The research simulated clinical implant site preparation under controlled laboratory conditions that reproduced key drilling parameters, including rotational speed, torque, and continuous irrigation. Four implant systems were evaluated: Osstem TSIII (Osstem Implant Co., Ltd., Seoul, Republic of Korea), Megagen AnyRidge (MegaGen Implant Co., Ltd., Daegu, Republic of Korea), Straumann BLT (Institut Straumann AG, Basel, Switzerland), and Bego RSX (BEGO Bremer Goldschlägerei Wilh. Herbst GmbH & Co., KG, Bremen, Germany).

- Each system included three sequential drills of increasing diameters (in millimeters), commonly used during implant bed preparation:
- Osstem: Os 2.0, Os 3.3, Os 3.5;
- Megagen: Me 2.0, Me 2.5, Me 3.3;
- Straumann: St 2.2, St 2.8, St 3.5;
- Bego: Be 2.0, Be 2.5, Be 3.25.

All drills were new and unused prior to the experiment. The length of each implant bed was 10 mm. Each drill type underwent a defined number of osteotomies under identical laboratory conditions, followed by surface evaluation using scanning electron microscopy (SEM) and a standardized wear scoring system.

### 2.2. Sample Preparation

The experimental model consisted of porcine rib bones (*Złotnicka Biała* breed) of comparable size with respect to length, thickness, and width. The mean diameter of the ribs used in this study was 18.23 ± 2.35 mm, while the average thickness of the cortical bone layer was 1.6 ± 0.7 mm.

**Figure 1:**
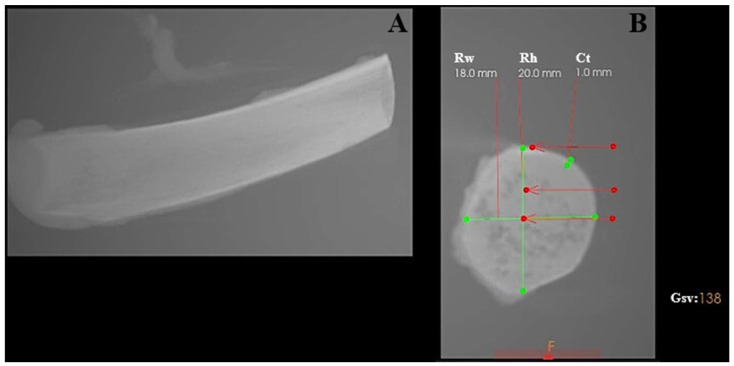
Characterization of the porcine rib used as the experimental bone model, analyzed using CS 3D Imaging software (version 3.10.48; Carestream Dental, Atlanta, GA, USA). (**A**) Longitudinal view of the porcine rib. (**B**) Cross-sectional view showing geometric measurements and grayscale-based bone density evaluation. Rw—rib width; Rh—rib height; Ct—cortical thickness; Gsv—grayscale value measured in the ventral part of the rib.

Bone density was assessed at different depths along the osteotomy site, reflecting the anatomical heterogeneity of the rib structure. In the cortical region, bone density measured in grayscale values corresponded to a range of approximately 700–1100 HU. At a depth of 5 mm, density values decreased to approximately 150–300 HU, while at 10 mm depth, values ranged between 50 and 150 HU, indicating predominantly trabecular bone structure (see Figure 1).

The porcine rib model was selected due to its combined cortical–cancellous architecture and is commonly regarded in implantology research as resembling medium-density human jawbone (D2–D3) rather than very dense cortical bone (D1) or highly trabecular bone (D4) [25].

For each implant system, ten osteotomies were performed on two ribs (five osteotomies per rib). Each experimental run therefore consisted of eight ribs (two ribs per system), resulting in a total of 40 osteotomies per run. This protocol was repeated three times using new sets of drills, yielding a total of 24 ribs and 120 osteotomies.

After each drilling series, all drills were carefully labeled and subjected to ultrasonic cleaning in distilled water to remove organic residues. Subsequently, the drills were sterilized in an autoclave at 134 °C for 10 min prior to SEM imaging to ensure surface cleanliness and to prevent contamination artifacts during microscopic examination.

### 2.3. Drilling Procedure

All osteotomies were performed using a NeoSurge implant micromotor (NeoBiotech, Seoul, Republic of Korea) equipped with a 32:1 reduction contra-angle handpiece. Drilling was conducted at a constant rotational speed of 1200 rpm under continuous irrigation with 4 °C sterile NaCl solution. A constant rotational speed of 1200 rpm was intentionally applied to all drills and implant systems to ensure standardized and reproducible experimental conditions. This approach was chosen to allow direct comparison of surface wear patterns between different drill designs and diameters, independent of manufacturer-specific drilling protocols. Therefore, the applied drilling speed does not aim to replicate individual IFU recommendations but serves a methodological standardization purpose. Our previous findings also support 1200 rpm with saline cooling at 4 °C as a parameter associated with reduced thermal and mechanical bone damage [8]. The applied axial pressure ranged from 0 to 720 g, depending on bone density and resistance. Before the experimental procedures, all drills were visually inspected under ×4.0 magnification to exclude pre-existing manufacturing defects and to confirm intact cutting edges and surface integrity. All procedures were performed by the same experienced operator to minimize operator-related variability.

### 2.4. SEM Imaging and Surface Analysis

Following sterilization, the working surfaces of all drills were examined using Scanning Electron Microscopy (SEM) (EVOMA25, ZEISS, Oberkochen, Germany) to assess microstructural wear and surface deformation. Imaging was performed at an accelerating voltage of 20 kV using a Backscattered Electron Detector (BSD, ZEISS, Oberkochen, Germany), which provides enhanced visualization of material-dependent contrast and microstructural defects. The probe current was set to 1.0 nA. All images were acquired at a fixed magnification of ×50, corresponding to a consistent scale bar of 200 μm across all images.

SEM micrographs were acquired from multiple orientations to provide a comprehensive and spatially accurate assessment of the geometry and condition of the cutting surfaces. The imaging angles were selected to capture complementary aspects of wear morphology and edge integrity:

- a vertical (axial) view,
- a horizontal (lateral) view, and
- a 45° oblique view.

Using this set of complementary views ensured that wear patterns were documented thoroughly and consistently, reducing the chance that any orientation-dependent damage would be missed.

### 2.5. Wear Assessment Criteria

Drill wear was evaluated using a seven-parameter descriptive scale, including:

1. Cleavage of the rake angle (CR),
2. Chipping at the corners (CC),
3. Chipping on the guide faces (CG),
4. Accumulation formation (CA),
5. Nicks on the main cutting edge (NC),
6. Nicks on the guide faces (NG),
7. Nursery wear (NW).

**Figure 2:**
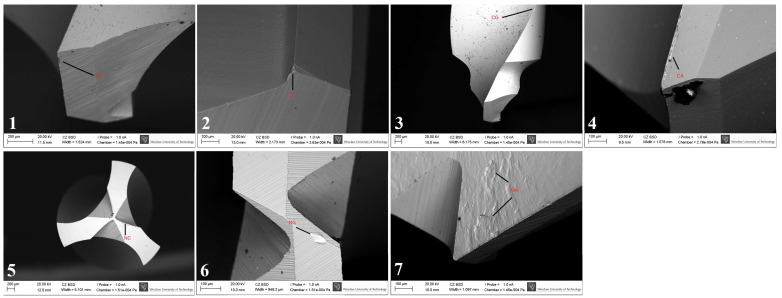
Representative SEM images illustrating the wear assessment criteria used in the descriptive scale. Individual panels show characteristic examples of cleavage of the rake angle (CR), chipping at the corners (CC), chipping on the guide faces (CG), accumulation formation (CA), nicks on the main cutting edge (NC), nicks on the guide faces (NG), and nursery wear (NW), as defined in Section 2.5.

Each parameter was scored on a binary scale (0 = absent, 1 = present). A total wear score was calculated as the sum of all positive findings for each drill (Figure 2).

### 2.6. Statistical Analysis

Multifactorial ANOVA with Tukey’s post hoc test was used to conduct statistical analyses and evaluate the effects of drill brand, drill diameter, and their interaction (brand × diameter) on the total wear score (TWS). To allow the use of drill diameter as an independent variable, the drills were categorized as small, medium or large. To evaluate the possible correlation between drill diameter and TWS, a Pearson correlation test was performed. For evaluation of possible wear patterns in different drills, hierarchical clustering was performed using the complete linkage method and the Euclidean distance metric. The analysis was performed using Jamovi 2.6 [39] and the R statistical environment v 4.4.2 [40] along with additional libraries [41,42,43]. The level of statistical significance was set at α = 0.01. TWS normality was verified using histogram inspection.

## 3. Results

### 3.1. Influence of Drill Brand and Diameter on Total Wear Score

**Figure 3:**
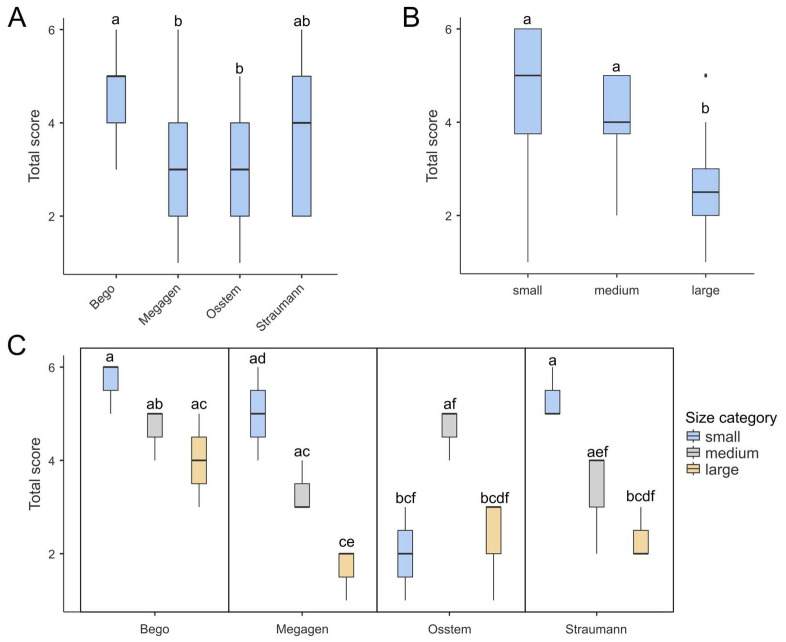
Box-plots demonstrating total score in for different (**A**) brands, (**B**) drill diameter and (**C**) combination of both. (**A**) Bego achieved the highest score, which was significantly higher than those of Megagen and Osstem. (**B**) Generally, large drills tend to achieve the lowest total score among all drill diameters. (**C**) Box plots presenting combinations of drill brands and diameters show one outlier: the small Osstem drill yielded a notably low score, despite the fact that low-diameter drills generally tend to score highly. Each box shows the interquartile range (IQR) and the median marked by a bold line. Whiskers show the 1.5 IQRs, dots indicate outliers. Boxes labeled with at least one same lowercase letter (a–f) show no statistically significant differences (post hoc Tukey test, α = 0.01).

**Figure 4:**
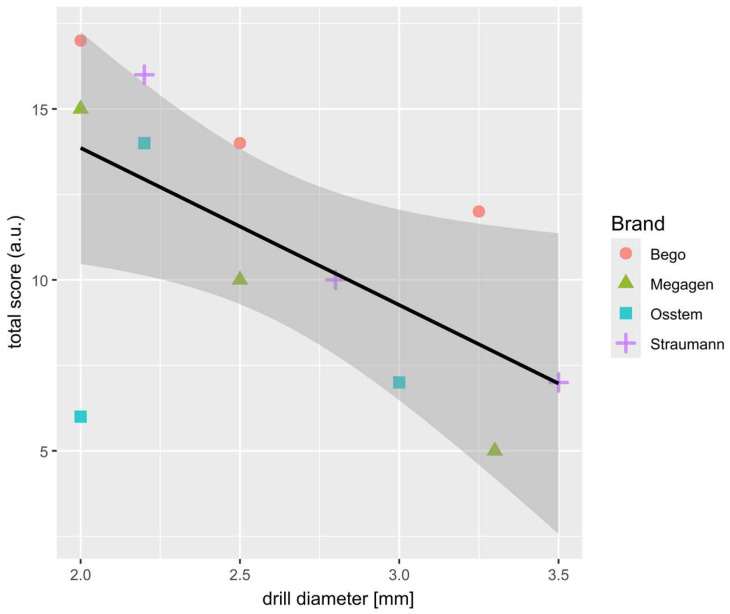
Scatter-plot showing the strong correlation between the sum of TWS (from 3 drill of the same brand and diameter) and drill diameter. Results for each brand are marked with different colors and shapes. Most outlying results (2.0; 6.0) indicate the Osstem 2.0 mm drill.

Multifactorial ANOVA revealed that both the drill brand and drill diameter, as well as their interaction (brand × diameter), significantly influenced the total wear score (TWS) (Table 1). All factors were statistically significant (*p* < 0.001), with effect sizes (η^2^) ranging from 0.20 to 0.30, indicating a moderate association between these variables and the degree of surface wear. As shown in Figure 3A, Bego drills achieved the highest mean TWS, significantly exceeding those of the Megagen and Osstem systems (*p* < 0.01, Tukey post hoc test). Figure 3B demonstrates a clear dependence of wear on drill diameter: smaller drills exhibited the highest TWS, whereas larger drills showed markedly reduced wear. When both factors were combined (Figure 3C), one distinct outlier emerged—the Osstem 2.0 mm drill, which displayed an unusually low TWS despite its small diameter. This anomaly was also evident in the correlation plot (Figure 4). The results indicated that both the material and geometry of the drill, as well as its working diameter, have a measurable effect on the extent of surface degradation observed after repeated osteotomies.

### 3.2. Relationship Between Drill Diameter and Total Wear Score

A strong negative correlation was observed between TWS and drill diameter, confirming that smaller-diameter drills are more susceptible to wear. This trend reflects the higher mechanical load and friction experienced by pilot drills during initial cortical bone penetration. The Osstem 2.0 mm drill again deviated from this general pattern, exhibiting an unexpectedly low TWS, which may be attributed to its specific cutting geometry and lower conicity, reducing contact stress during bone entry. The relationship between drill diameter and overall surface wear is further illustrated in Figure 4.

### 3.3. Analysis of Wear Patterns Based on Clustered Heatmap

**Figure 5:**
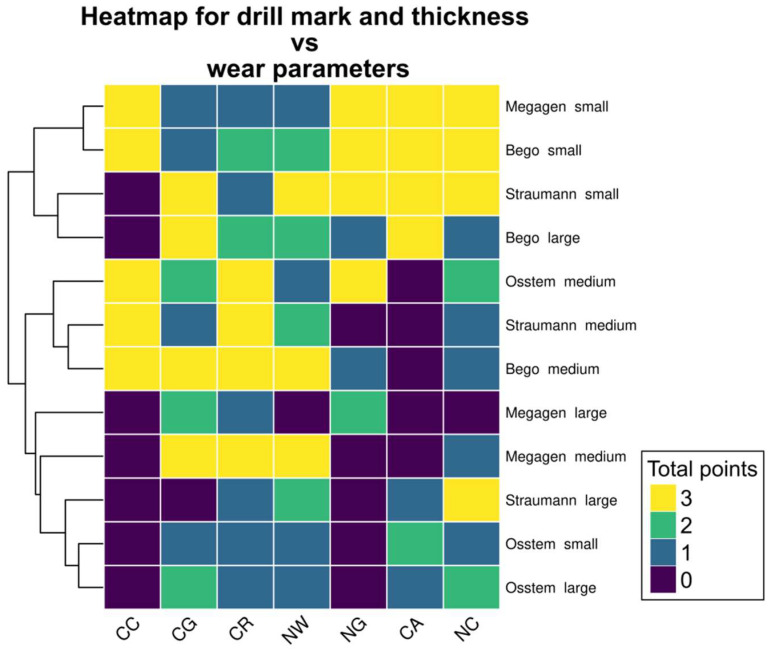
Heatmap showing the possible patterns of different drill wear. As three sets of drills were used, the maximum wear score in each category is 3 and the minimum is 0. Small drills (except Osstem) scored highly in NG, CA and NC, while medium drills—in CC, CG, CR and NW. Large drills (except for Bego large drill) had a low overall score in all evaluated categories. Notably, the CC wear score was either 3 or 0; this parameter achieved constant score in all repetitions.

While the total wear score provides a global measure of drill degradation, it does not differentiate among specific wear characteristics. To identify potential wear trends, a clustered heatmap was constructed based on seven evaluated parameters (Figure 5).

Three distinct clusters were identified, corresponding broadly to drill diameter categories. Small drills (except Osstem) displayed high wear intensity in nicks on guide faces (NG), accumulation formation (CA), and nicks on cutting edges (NC). Medium-sized drills demonstrated the greatest degradation in chipping at corners (CC), chipping on guide faces (CG), cleavage of the rake angle (CR), and nursery wear (NW). Large drills (except the largest Bego drill) showed generally low wear in all categories, with NG and CC parameters absent in most samples.

Notably, the CC parameter exhibited a binary pattern, scoring either 0 or 3 across repetitions, suggesting that this form of damage either consistently occurred or was entirely absent depending on the drill type. These patterns emphasize the non-random distribution of wear phenomena and suggest that different drills are predisposed to specific modes of surface degradation depending on their geometry and manufacturing characteristics.

### 3.4. SEM Surface Characterization

**Figure 6:**
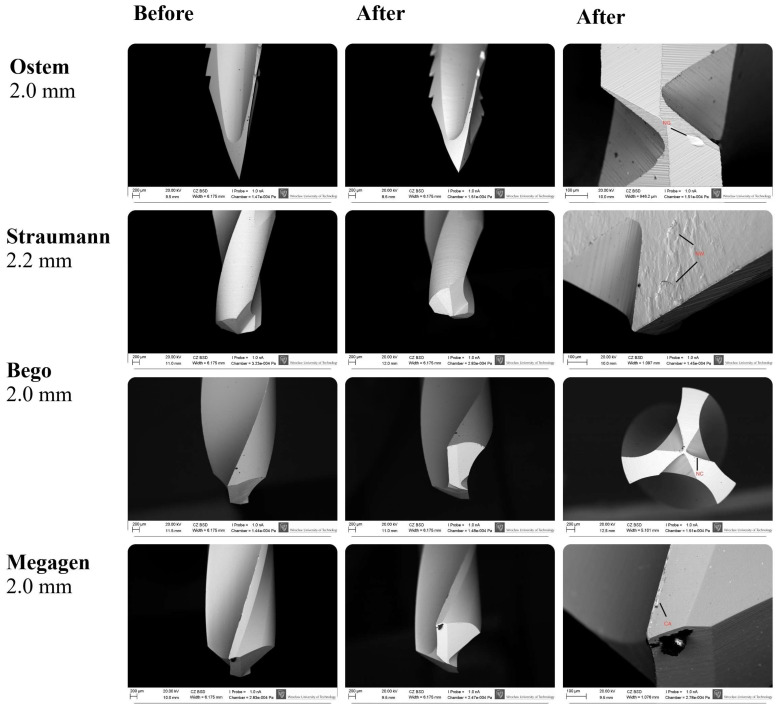
SEM images showing the surface morphology of Osstem, Megagen, Straumann, and Bego pilot drills before and after simulated clinical use. Nominal drill diameters (mm) are indicated. Red labels denote characteristic wear features: CA—accumulation formation; NC—nicks on the main cutting edge; NG—nicks on the guide faces; NW—nursery wear. Images were acquired at varying magnifications (≈×30–300; scale bars: 200 µm and 100 µm). Apparent differences in drill tip size reflect variations in drill geometry, cutting-edge design, imaging orientation, and field of view rather than nominal diameter. The images shown are representative examples selected from multiple SEM observations performed at different orientations and magnifications, as described in Section 2.4.

SEM analysis revealed that the highest degree of surface deformation occurred in the pilot (small-diameter) drills across all implant systems, which is consistent with the increased mechanical load applied during initial cortical penetration. In contrast, larger-diameter drills—primarily used for osteotomy enlargement—exhibited visibly reduced wear due to operating under lower cutting resistance. Among the pilot drills, Osstem instruments showed the least surface degradation, maintaining sharp cutting edges and presenting minimal chipping across all examined viewpoints. Bego drills demonstrated the greatest degree of microdamage, characterized by more pronounced chipping, localized surface defects, and irregularities along the cutting edges. Megagen drills showed the most uniform and smooth surface morphology, with fewer microfractures and abrasion patterns compared with the other systems. Straumann drills displayed moderate level of wear, predominantly expressed as edge rounding and minor abrasive markings. Representative SEM images illustrating the characteristic wear patterns are presented in Figure 6. It should be emphasized that Figure 6 includes selected micrographs for illustrative purposes. The qualitative wear assessment was based on the evaluation of multiple SEM images acquired from each drill at different orientations and viewing angles, as well as at varying magnifications, in accordance with the methodology described in Section 2.4, rather than on single imaging locations.

## 4. Discussion

The aim of the present study was to evaluate the degree and pattern of wear occurring on the working surfaces of implant drills subjected to repeated osteotomy cycles under standardized laboratory conditions. The results clearly demonstrated that drill wear was significantly influenced by both drill brand and diameter, with the highest degradation observed in small-diameter pilot drills and the lowest in large-diameter instruments. Among the assessed systems, Bego drills showed the greatest overall wear, whereas Osstem drills—particularly the 2.0 mm pilot drill—displayed unexpectedly low surface degradation despite their small size. These findings correspond with previous reports indicating that drill geometry, surface coatings, and microstructural properties significantly influence wear resistance and cutting performance [3,36,44]. In the context of dental implantology, drill wear represents a crucial factor affecting both surgical efficiency and biological outcomes. Numerous studies have shown that repeated use and sterilisation lead to progressive degradation of cutting surfaces, increased friction, reduced cutting efficiency, and elevated heat generation during osteotomy [6,8,36,45]. However, in the present study no direct measurements of temperature, torque, or axial force were performed; therefore, references to thermal effects represent inferences based on the published literature rather than outcomes directly assessed in this experiment. Excessive thermal accumulation poses a significant biological risk, as temperatures exceeding 47 °C sustained for more than one minute may induce osteocyte injury, microcrack formation, and compromised osseointegration [32,33,34,46]. Additionally, systematic reviews have highlighted that conventional rotary drilling can induce microdamage—such as irregular osteotomy margins and localized thermal injury—if parameters such as irrigation, drill geometry, and rotational speed are not optimally controlled [9]. It should be emphasized that rotational speed is a critical yet highly variable parameter in clinical implantology, as manufacturers provide system- and diameter-specific recommendations intended to optimize cutting efficiency and minimize thermal and mechanical damage [9]. As a result, no universal drilling speed protocol exists, and direct comparison between implant systems operating under manufacturer-recommended conditions is inherently challenging. This variability underscores the importance of standardized experimental conditions when comparative assessments of drill wear are performed [9,10]. Therefore, monitoring drill condition and adhering to manufacturer recommendations regarding reuse cycles are essential steps in maintaining surgical safety.

Bone trauma resulting from mechanical osteotomy is a multifactorial process that directly affects implant stability. Rotational speed does not act as an isolated determinant of biological outcome, but rather interacts with axial load, drilling time, irrigation efficiency, and the degree of drill wear [22,47,48]. Mechanical drilling may induce both thermal and mechanical injury depending on rotational speed, axial load, irrigation quality, and the degree of drill wear [8,49]. Excessive friction or insufficient cooling can cause carbonization, microcracks, and distortion of bone microarchitecture, further exacerbated when worn drills are used [8]. Documented mechanical trauma includes microfractures at osteotomy margins, compressed or distorted lamellae, and irregular bone debris, all of which negatively influence early healing and bone–implant contact [50,51,52,53,54]. Experimental studies have demonstrated that lower rotational speeds may prolong drilling time and increase frictional contact, whereas excessively high speeds—particularly when combined with insufficient load control—may also elevate friction and heat generation [10,22,47,48]. Consequently, optimal drilling performance depends on a balanced interaction between speed, force, and cutting efficiency rather than on rotational speed alone [10,22,47,48]. Higher rotational speeds combined with increased axial force have been reported to enhance cutting efficiency compared with low-speed drilling, thereby limiting friction-related heat generation. Brisman [48] demonstrated that drilling performed at 2400 rpm under a load of 2.4 kg resulted in lower temperature elevation than drilling at 1200 rpm with a lighter load of 1.2 kg, emphasizing the importance of the interaction between speed and applied force rather than rotational speed alone. Hobkirk and Rusiniak [55] identified approximately 1.2 kg as the average force applied to the handpiece during routine osteotomy, although thermal outcomes were not evaluated, while Cordioli and Majzoub [56] reported that drilling forces of around 2 kg fall within the range commonly used under clinical conditions. These findings are consistent with our previous experimental investigations, which demonstrated that an intermediate drilling speed of 1200 rpm represents an optimal balance between cutting efficiency and thermal safety [10]. In an in vitro study, drilling at 800 rpm resulted in prolonged preparation time, increased friction, and higher heat accumulation, whereas drilling at 1500 rpm led to excessive rotational friction, also increasing thermal load [10]. These results indicated that while manufacturer recommendations for drill speed may vary according to system and diameter, a rotational speed of approximately 1200 rpm frequently represents a biomechanically and thermally favorable compromise when adequate cooling and controlled drilling force are applied.

To enhance durability and reduce friction, manufacturers increasingly introduce modifications to drill geometry and utilize advanced surface coatings. Both macro-design and micromorphology strongly influence cutting force and wear tendency [3]. Protective coatings such as titanium–aluminum nitride, diamond-like carbon, or boron-based films have demonstrated the ability to reduce friction, lower temperature generation, and significantly extend drill lifespan [38]. Furthermore, zirconium oxide drills have emerged as a promising alternative to conventional steel drills due to their substantially higher resistance to wear and more stable cutting-edge morphology under repeated use [7]. In recent years, alternative osteotomy methods—particularly piezoelectric surgery and laser-based osteotomy—have been proposed to reduce the mechanical and thermal trauma associated with rotary drilling. Piezosurgery enables micrometric, selective bone cutting with fewer microcracks, improved healing, and reduced postoperative discomfort, although the technique requires more operative time [57]. Similarly, Er:YAG lasers offer precise, minimally traumatic bone cutting due to energy absorption in water-rich tissues, thereby reducing carbonization and promoting more favorable early healing [58]. Despite these advantages, limitations such as slower cutting speeds and challenges in controlling depth and angulation limit their widespread adoption. Nevertheless, these technologies represent important directions toward biologically safer implant bed preparation.

This study has several limitations. Most importantly, a single rotational speed was applied across drills of different diameters, which does not fully reflect manufacturer-specific clinical recommendations. While this approach enabled standardized and controlled comparison of wear patterns between systems, it may have influenced the magnitude and characteristics of observed surface degradation. The use of porcine ribs, although comparable to human bone in structure, does not fully replicate the diversity of human cortical density and anatomy. No direct measurements of thermal output, torque, or axial force were performed, which limits the ability to establish causal relationships between drill wear and biological or mechanical consequences. The limited number of osteotomies performed with each drill does not simulate extended clinical reuse or multiple sterilisation cycles. Additionally, drills were sterilized only after short series of osteotomies, and the cumulative effects of prolonged clinical use and repeated sterilization—common in practice—were not evaluated. These aspects should be considered when interpreting the results. Although SEM provided detailed morphological insight, the analysis was qualitative, and future studies may benefit from quantitative evaluation such as 3D profilometry or micro-CT. Future research should incorporate diameter-specific drilling speeds, clinically realistic drilling forces, and integrated thermal measurements to better reflect real-world surgical conditions. Integrating mechanical, thermal, and biological outcomes—such as osseointegration quality—will deepen understanding of how drill wear affects clinical performance. Ultimately, combining laboratory results with in vivo validation may contribute to the development of improved drilling protocols and next-generation osteotomy tools enhancing safety and long-term implant success.

## 5. Conclusions

This in vitro study demonstrated that the extent and characteristics of drill wear during implant site preparation are influenced by both drill brand and diameter. Small-diameter pilot drills exhibited the highest degree of surface degradation, whereas larger drills showed markedly lower wear. Among the evaluated systems, Bego drills presented the greatest overall wear, while Osstem drills—particularly the 2.0 mm instrument—displayed unexpectedly low degradation despite their small size. SEM analysis revealed distinct patterns of edge rounding, chipping and guide-face damage, confirming that repeated osteotomies lead to progressive microstructural deterioration. The findings of the present study emphasized that drill wear is not a uniform process but depends on material properties, surface coatings, cutting geometry and chip evacuation efficiency. As wear increases, the risk of friction-induced thermal elevation also rises, potentially exceeding thresholds associated with bone necrosis and impaired osseointegration. These results underline the clinical importance of monitoring drill condition, adhering to recommended reuse limits and considering the use of drills with enhanced coatings or wear-resistant materials. Future research integrating real-time thermal and mechanical measurements, extended drilling cycles and in vivo validation will be crucial for developing evidence-based guidelines on drill lifespan and improving the safety and predictability of implant site preparation.

## Figures and Tables

**Table 1:** Results for multifactorial ANOVA for evaluation of influence of distinct parameters on total wear score. All parameters (as well as their combination) considered in the current study had significant influence on it (*p* < 0.001). η^2^ indicates the strength of association between a variable and the observed outcome; the results in the range 0.2–0.3 suggest a fair association.

	Sum of Squares	df	Mean Square	F	*p*	η^2^
Brand	16.1	3	5.361	8.04	<0.001	0.202
Thickness	23.7	2	11.861	17.79	<0.001	0.298
Brand × thickness	23.8	6	3.972	5.96	<0.001	0.299
Residuals	16.0	24	0.667			

## Data Availability

The original contributions presented in this study are included in the article. Further inquiries can be directed to the corresponding authors.
